# Differences in the Chromogenic Effect of Corn Starch and Potato Starch on Paprika Red Pigment and Structural Characterisation

**DOI:** 10.3390/foods13020191

**Published:** 2024-01-06

**Authors:** Fan Su, Yongqiang Wu, Yanping Cao, Shaojia Wang

**Affiliations:** Beijing Advanced Innovation Center for Food Nutrition and Human Health (BTBU), School of Food and Health, Beijing Higher Institution Engineering Research Center of Food Additives and Ingredients, Beijing Technology and Business University, Beijing 100048, China; sf19980817@163.com (F.S.); wuyongqiang0095@163.com (Y.W.); caoyp@th.btbu.edu.cn (Y.C.)

**Keywords:** corn starch, potato starch, paprika red pigment, chromogenic effect, structural characterisation

## Abstract

The present study aims to investigate the chromogenic effect and the interaction between starch-pigment complexes of corn starch (CS) and potato starch (PS) complexed with paprika red pigment. Compared to PS, CS showed 12.5 times higher adsorption capacity for paprika red pigment. Additionally, the a* value of CS-P (26.90 ± 0.23) was significantly higher than that of PS-P (22.45 ± 1.84), resulting in a corn starch-paprika red pigment complex (CS-P) with a more intense red colour. The addition of paprika red pigment significantly decreased the particle size and porosity of CS by 48.14 ± 5.29% and 17.01 ± 3.80%, respectively. Conversely, no significant impact on PS was observed. Additionally, the Fourier transform infrared (FT-IR) spectroscopy results revealed that the starch molecules and paprika red pigment were bound to each other through strong hydrogen bonds. X-diffraction (XRD) results indicated that the starch-paprika red pigment complexes have a V-shaped structure. Furthermore, the relative crystallinity of the complexes between starch and red pepper pigment showed an increasing trend, however, the relative crystallinity of CS increased significantly by 11.77 ± 0.99–49.21 ± 3.67%. Consequently, the CS-P colouring was good.

## 1. Introduction

Paprika red pigment, consisting of capsorubin, capsanthin, β-carotene, zeaxanthin, and β-cryptoxanthin, is a high-quality natural dye mainly extracted from dried chili peppers [1]. As a precursor of vitamin A, paprika red pigment possesses various physiological functions. Studies have shown that paprika red pigment has the potential to regulate lipid metabolism, thereby reducing the risk of cardiovascular diseases, cancer, and other chronic diseases [2]. Paprika red pigment has been commercialized as colourants, feed supplements, and nutritional products due to its wide distribution, bright colour, high safety, and anti-oxidation, and is widely used in food, medical, feed, cosmetics, and other fields [3]. For instance, paprika red pigment imparts higher initial redness to fresh red sausage and fresh chorizo (red-line meat products), which remains unchanged until the end of the product’s shelf life [4]. Paprika red pigment could ameliorate the detrimental effects of diet-induced obesity by improving impaired lipid metabolism [5].

With the emerging growth of the social economy, people’s consumption level has increased considerably, accelerating the demand for food with high standards for appearance and sensory quality and meeting healthier dietary requirements. Studies have indicated that red environments are often more stimulating for consumers, leading to an augmented interest in purchasing products. Hence, developing products with appealing colours and appearance is crucial within the food sector. The food industry has been working on the development of highly stable, attractive colours, and low-cost synthetic dyes to improve the appearance of food products. Nevertheless, synthetic food additives, such as carmine and quinoline yellow, have the potential to cause health issues such as allergies, irritability, and even more severe conditions like cancer. Therefore, in recent years, research on the development and application of natural dye has received considerable attention [6]. Although paprika red pigment has a wide range of applications in the food industry, its high lipophilicity, low water solubility, and light sensitivity significantly hinder its application as a natural food additive [7]. To address these issues, efforts were made to encapsulate paprika red pigment in lipid nanoparticles, liposomes, and emulsions [8]. Paprika red pigment may also interact with proteins [9], polysaccharides [10], and other inorganic nanoparticles [11] to form complexes with high stability and activities. Particularly, polysaccharides are promising delivery systems due to their good biocompatibility and biodegradability [12]. For example, in a previous study, the stability of the colour index in yoghurt stained with the complex formed by paprika red pigment and β-cyclodextrin was higher than that of yoghurt stained with paprika red pigment alone [13]. Additionally, the capsaicin-chitosan colloidal complex had high colloidal stability and high apparent solubility [14]. Ethyl cellulose-capsaicin composite membrane has the advantages of ease in production, eco-friendliness, and high antimicrobial activity [10]. Therefore, polysaccharides can be used as potential carriers of natural pigments such as paprika red pigment with wide research prospects.

Starch, derived from the polymerisation of glucose molecules, is a polysaccharide widely used in food as a good wall material. It is reported that the interaction of β-carotene and sweet potato starch could improve the bioaccessibility and bioavailability of β-carotene and starch molecules [15]. In a previous study, a curcumin emulsion delivery system was developed using debranched starch as a carrier, which offered improved stability and solubility of curcumin compared to the system developed using Tween 80 and lectin [16]. CS can be used as a carrier for grape seed proanthocyanidins [17]. Seaweed polyphenols bind to CS and promote its gelation [18]. Starches and pigments are mainly connected by non-covalent bonds [19]. A previous study on the interaction between sorghum proanthocyanidin and amylose and demonstrated that sorghum proanthocyanidin interacts effectively with amylose through hydrophobic and hydrogen bonding [20]. The study discovered that lutein binds to soybean starch through hydrophobic forces, resulting in a complex that significantly enhances lutein stability [21]. The tremella fuciformis polysaccharide could interact with PS mainly through hydrogen bonds [22]. The barley β-glucan interacts with PS through hydrogen bonding, thereby improving the gelling properties of PS [23]. To date, some interactions of capsaicin with indica starch and high-amylose corn starch have been reported internationally [24,25]. However, no studies have reported the complex mechanisms between paprika red pigment and corn starch (CS), the most dominant in the starch industry, and between paprika red pigment and potato starch (PS), the second most important starch in the industry. Therefore, in this study, corn starch-paprika red pigment (CS-P) and potato starch-pigment (PS-P) complexes were prepared from paprika red pigment, CS, and PS, and their physicochemical properties were characterised using scanning electron microscopy (SEM), pore space, high-performance liquid chromatography (HPLC), particle size, Fourier transform infrared spectroscopy (FT-IR), and x-ray diffraction (XRD) technique. Furthermore, the adsorption capacity and colour rendering effect of CS and PS on paprika red pigment were determined. The experimental results provide a theoretical basis for developing a natural colourant that can be used as a substitute for nitrite.

## 2. Materials and Methods

### 2.1. Materials

Paprika red pigment E150 (>98%) was provided by M&G Biotechnology Group Co., Ltd. (Handan, China). Corn starch (>99%) was purchased from Yishui Dadi Corn Development Co., Ltd. (Yishui, China). Potato starch (>99%) was purchased from Yantai Shuangta Food Co., Ltd. (Yantai, China). Amylopectin was obtained from Shanghai Aladdin Biochemical Technology Co., Ltd. (Shanghai, China). Amylose was purchased from Sigma-Aldrich Co., Ltd. (St. Louis, MO, USA). Methanol (MeOH) (Chromatographic grade) and methyl tert-butyl ether (MTBE) (Chromatographic grade) were purchased from Beijing Mairuida Technology Co., Ltd. (Beijing, China). Capsanthin standard sample (>97%), capsorubin standard sample (>97%), zeaxanthin standard sample (>97%), and β-cryptoxanthin standard sample (>97%) were obtained from Shanghai Huicheng Biotechnology Co., Ltd. (Shanghai, China). β-Carotene standard sample (>97%) was purchased from Shanghai Aladdin Biochemical Technology Co., Ltd. (Shanghai, China). The reagents acetone, n-hexane, and ethyl ether were of analytical grade and were purchased from Sinopharm Group Chemical Reagent Co., Ltd. (Shanghai, China). Sodium chloride, anhydrous sodium sulfate, sodium hydroxide, potassium iodide, and iodine were purchased from Fuchen (Tianjin) Chemical Reagent Co., Ltd. (Tianjin, China).

### 2.2. Preparation of Starch-Paprika Red Pigment Complexes

Preparation of starch-paprika red pigment complexes was performed as follows: First, 2 g of different types of starch (corn starch, potato starch, amylose, and amylopectin) and 50 mg paprika red pigment were mixed with 28 mL of MeOH to make a starch-paprika red pigment suspension. Then, the starch-paprika red pigment suspension was heated at 70 °C (DF-101S, Gongyi Yuhua instrument Co., Ltd., Gongyi, China) for 30 min with continuous stirring and then cooled to indoor temperature. Subsequently, the starch-paprika red pigment suspension was centrifuged at 4 °C and 8000 r/min (JXN-30, Beckman Coulter Co., Ltd., Brea, CA, America) for 20 min, and the precipitate was collected. The precipitate was washed twice with MeOH to remove the free material that was not bound to the starch. Finally, the starch-paprika red pigment complexes were recovered by drying in a vacuum freeze dryer (LGJ-1C-56, Beijing Yatai Colon Instrument Technology Co., Ltd., Beijing, China).

### 2.3. Colour Evaluation of Starch-Paprika Red Pigment Complexes

Colour values (L*, a*, b*) of PS-P and CS-P were determined via a spectrophotometer (CR-800, Beijing Kemerunda Instruments Co., Ltd., Beijing, China). The hue (h) of samples were calculated using the following equation [26],

$$h=\tan^{-1}\frac{b^{*}}{a^{*}}$$

where L, a, and b represent the sample brightness, red/green value, and yellow/blue value, and L*, a*, and b* represent the control brightness, red/green value, and yellow/blue value.

### 2.4. Determination of Reflectance and Scattering Rate of Starch-Paprika Red Pigment Complexes

The starch-paprika red complexes were placed in a quartz cuvette measuring 1 cm, and their reflectance was measured using the reflectance mode of the spectrophotometer (CR-800, Beijing Kemerunda Instruments Co., Ltd., Beijing, China) within a range of 400–700 nm. The transmission mode of the spectrophotometer was utilized to determine the transmittance and haze of the samples, and based on the following equation, the scattering rate of the starch-paprika red pigment complexes was calculated.

$$Scattering\;rate\left(\%\right)=transmittance\times haze$$

### 2.5. Analysis of Pigments Adsorbed by Starch in Paprika Red Pigment

#### 2.5.1. Pretreatment of Samples

Using the method by Kim et al. [27], appropriate adjustments were made to perform the following analysis. For the analysis of carotenoids, extraction was carried out using acetone. For the extraction, 1 g of dried sample was mixed with 20 mL of acetone and shaken for 20 min, and was repeated until the solution was colourless. The supernatants were merged and the solvents were evaporated to dryness using a rotary evaporator (RE-52AA, Shanghai Yarong Company Biochemical Instrument Factory, Shanghai, China) at 35 °C, dissolved with 15 mL ether/n-hexane [1:1(*w*/*w*)], then incubated with 15 mL MeOH and 5 mL 30% KOH/MeOH at room temperature for 2 h 30 min in the dark, and this was repeated until the solution was colourless. The supernatants were then washed several times with distilled water until the pH was neutral, and 5 mL of 10% NaCl and 5 mL of 2% Na_2_SO_4_ were added and discarded as the separated hydrophilic phase. After evaporating the collected extracts, the residue was dissolved in MeOH/MTBE (1 mL) [1:1(*w*/*w*)] and passed through a 0.22 μm microporous filter membrane until ready for use. Standard curves were plotted using standards of capsorubin, capsanthin, zeaxanthin, β-cryptoxanthin, and β-carotene that were >97% pure. The calculation of the adsorption ratio of starch to paprika red pigment was performed utilizing the subsequent formula,

$$Adsorption\;ratio\left(\%\right)=\frac{S}{S_{Total}}\times 100\%$$

where S represents the content of individual pigments in the starch-paprika red complexes and Stotal represents the total pigment content in the starch-paprika red complexes.

#### 2.5.2. Analysis of Pigments Content in CS-P and PS-P

The content of five pigments in CS-P and PS-P complexes was determined via HPLC (Waters 2695, Waters Corporation, Milford, MA, USA). Using the method by Murillo et al. [28], with some modifications, separation was performed using a Venusil XBP C30 column (4.6 × 250 mm, 5 μm) (Tianjin Bona Ijar Technology Co., Ltd., Tianjin, China) at 25 °C, and the mobile phase was a binary solvent consisting of phase A (MeOH/MTBE/W, 81:15:4, *v*/*v*/*v*) and phase B (MeOH/MTBE/W, 6:90:4, *v*/*v*/*v*). The UV wavelength was set to 450 nm. The injection volume was set to 1 μL and the flow rate was 1 mL/min. The gradients were programmed as follows (%B): first, 0–45 min, 0–50% of B; second, 45–50 min, 50–0% of B; and finally, 50–60 min, 0% of B.

### 2.6. Determination of Complexing Index (CI) of Samples

The values of *CI* of CS-P, PS-P, amylose-P, and amylopectin-P were measured using the method described by Wang et al. [29] with a slight modification. Briefly, 1 mL of the samples mentioned above were mixed with 200 µL I2/KI [1.3% (*w*/*w*) I2 and 2.0% (*w*/*w*) KI in deionized water] solution, and the absorbance was measured at 620 nm via a Ultraviolet-visible Spectrophotometer (SHIMADZU-1280, Shimadzu Corporation, Kyoto, Japan). The complex index was calculated using the following equation [30].

$$CI\left(\%\right)=\frac{{Abs}_{starch}-{Abs}_{starch-paprika\;red\;pigment}}{{Abs}_{starch}}\times 100\%$$

### 2.7. Scanning Electron Microscopy (SEM) Analysis 

The microscopic morphology of the pretreated samples was observed using a S4800 SEM (Hitachi Production Co., Ltd., Tokyo, Japan). The dried samples were mounted on a copper stake with a double-sided carbon tag and coated with platinum for 80 s. SEM images were taken at an accelerating voltage of 15 kV and captured using the accompanying software (Digital Micrograph 3.4).

### 2.8. Particle Size Analysis 

The suspended particle sizes of PS, CS, PS-P, and CS-P were determined via particle size analyzer (Mastersizer 2000, Malvern UK Go., Ltd., London, UK). The optical path of the cuvettes used was 1 cm, and the refractive index and viscosity of the samples were calculated using standard calculation tools provided by the software. During the process, the sample was balanced for at least 1 min, and at least 13 consecutive readings were taken. 

### 2.9. Mercury Porosimetry Analysis

Using the method by Włodarczyk-Stasiak et al. [31], the measurements were carried out using Mercury piezometers (AutoPore V 9600, Micromeritics Corporation, Norcross, GA, USA). Briefly, 1 g of starches and starch-capsaicin complexes was dried at 105 °C, placed in a dilatometer, outgassed underhigh vacuum, and filled with mercury under a pressure range of 0.1 to 61,000 psi.

### 2.10. Fourier Transform Infra Red (FT-IR) Spectroscopy

The FT-IR spectrometer (IS 5, Thermo Fisher Scientific, Waltham, MA, USA) was used to scan the FT-IR spectra of PS, CS, PS-P, and CS-P at full wavelength (4000–400 cm^−1^) at room temperature. The dried samples mentioned above and 100 mg KBr were mixed in the mixing mortar to make the powder more uniform. The spectrum was recorded using 32 scans with a resolution of 4 cm^−1^. Each sample was measured 3 times in parallel.

### 2.11. X-ray Diffraction

X-ray diffraction analyses of PS, CS, PS-P, and CS-P were performed using a SE diffractometer (Nihon Riken Electric Co., Ltd., Niigata, Japan) operated at 40 kV and 40 mA with graphite-filtered Cu Ka radiation and a q compensating slit. The relative intensity was recorded in a scattering angle range (2θ) of 5–60° with a scintillation counter at a scanning speed of 0.02°/min, 17.7 s/step.

### 2.12. Statistical Analysis

Results are shown as mean ± standard deviation (*n* = 3). Statistical analysis was carried out using SPSS System Software 22.0. Significant differences between the individual means were tested using *t*-test and Duncan’s multiple range (*p* < 0.05). All graphs involved in this article were drawn using OriginLab 2021 software, and all tables were drawn using Office Word 2019 software.

## 3. Results

### 3.1. CS-P and PS-P Colour Comparison

**Figure 1:**
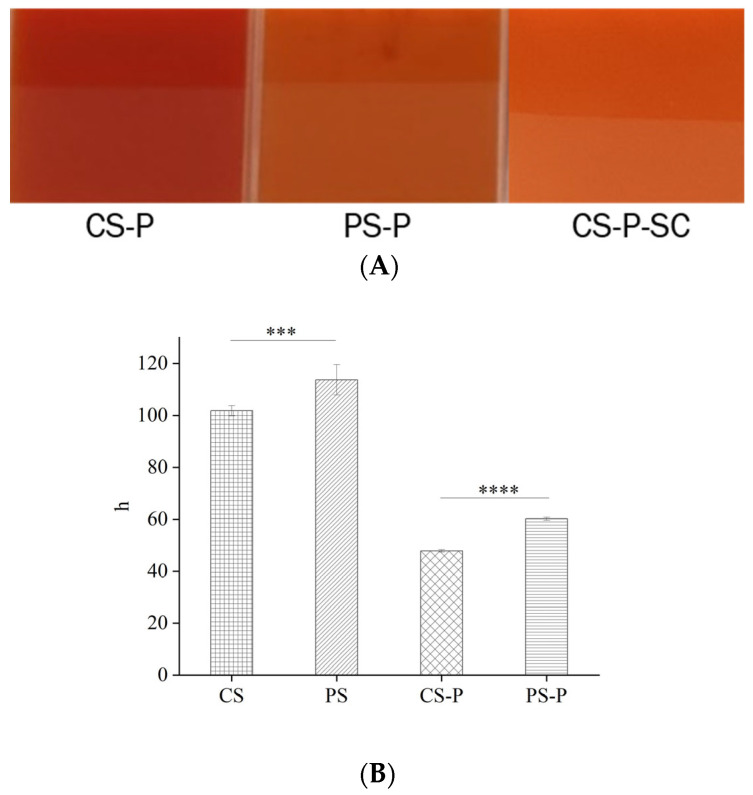
Comparison of the colour rendering effect of starch-paprika red pigment complexes (**A**) and h-values of starches and starch-paprika red complexes (**B**) are shown. CS: Corn starch, PS: Potato starch, CS-P: Corn starch-paprika red pigment, PS-P: Potato starch-paprika red pigment, and CS-P-SC: Corn starch-paprika red pigment-sodium caseinate. Significant difference was defined as *p* < 0.05 (*** *p* < 0.001, **** *p* < 0.0001).

The interactions between CS and PS with paprika red pigment were assessed using a binary mixed system consisting of starch-paprika red pigment complexes. Figure 1A displays the adsorption capacity of CS and PS for paprika red pigment. The results showed that there was a significant difference in the adsorption capacity of CS and PS for paprika red pigment, with CS-P being more reddish. Colour space is a geometric representation of colour in three dimensions, which can be calculated and derived from the initial stimulus values of red, green, and blue. Chromatic aberration is a viable measure to assess the extent of colour variation between different substances [32]. Therefore, in this study, the L*, a*, and b* values were used to verify the colour differences among the samples. The CS-P L*, a*, and b* values exceeded those of PS-P (Table 1), and the total colour difference ΔE was considerably higher than that of PS-P. This outcome is consistent with the findings in Figure 1A. The hue (h) is a qualitative characteristic of colour, traditionally defined as the property of red and green. It provides a clearer indication of the degree to which starch is bound to pigments, proteins, polyphenols, and other substances [33]. As shown in Figure 1B, the h-value for PS was higher than that for CS, and both were greater than 90. Compared to CS and PS, the h-value for both CS-P and PS-P were significantly lower at 47.88 ± 0.50 and 60.22 ± 0.60, respectively, indicating that starches formed complexes with paprika red pigment and the binding capacity of CS for paprika red pigment was stronger than that of PS. Starch is formed by numerous polymer chains containing many hydroxyl groups and arrangements. The paprika red pigment also possesses many hydrophilic hydroxyl groups [34]. Therefore, it can be inferred from the above results that the interaction between starch macromolecules and paprika red pigment might be attributed to the hydrogen bonding or van deer Waals forces, facilitating the encapsulation of paprika red pigment into the starch particles [32].

### 3.2. Analysis of the Optical Properties of Starches and Starch-Paprika Red Pigment Complexes

**Figure 2:**
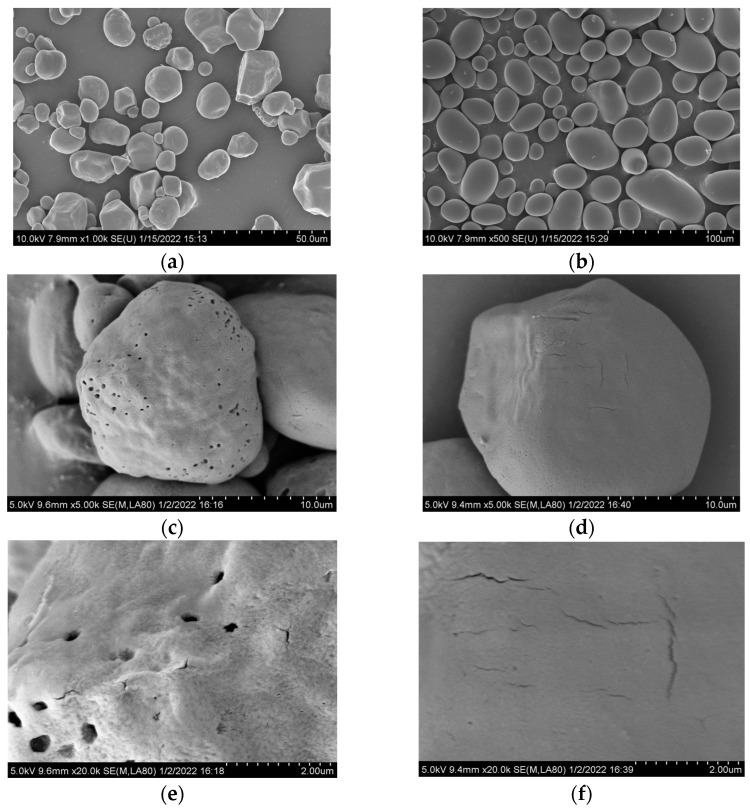
Microscopic morphology of CS (**a**,**c**,**e**) and PS (**b**,**d**,**f**), with ×1 k (**a**,**b**), ×5 k (**c**,**d**), and ×20 k (**e**,**f**) magnification. CS: corn starch, PS: potato starch.

**Figure 3:**
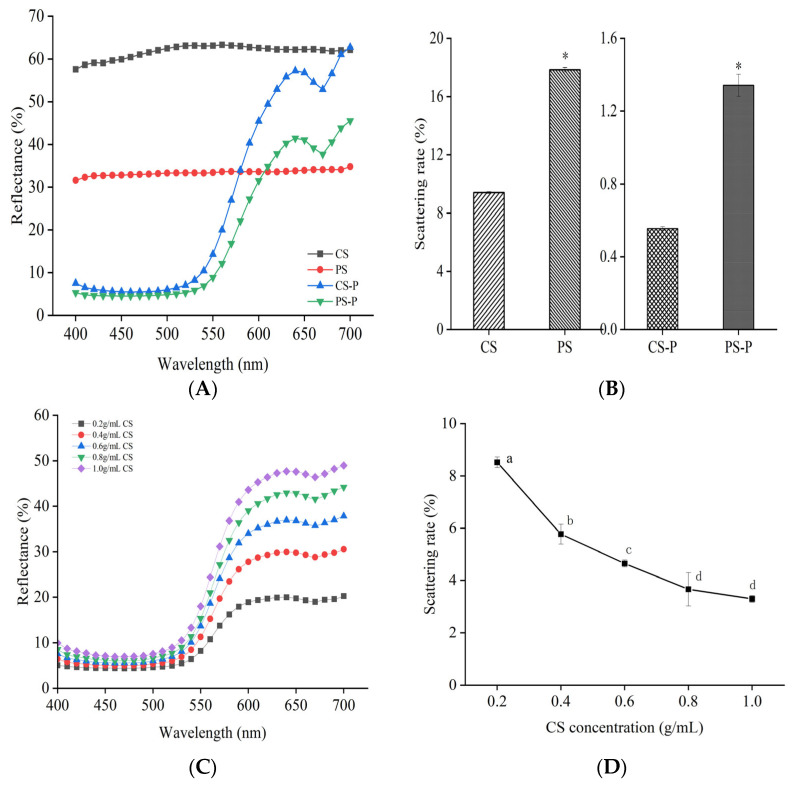
Reflectance spectra of starches and starch-paprika red pigment complexes (**A**), scattering rates of starches and starch-paprika red pigment complexes (**B**), effects of CS concentration on the reflection spectrum of CS-P (**C**), and effects of CS concentration on the scattering rate of CS-P (**D**) are shwon. CS: Corn starch, PS: Potato starch, CS-P: Corn starch-paprika red pigment, and PS-P: Potato starch-paprika red pigment. Significant difference was defined as * *p* < 0.05. Different letters indicate significant differences (*p* < 0.05).

Reflectance and scattering could describe the different phenomena occurring on the surface of an object and are used to describe the interaction between light and the object. For most substances, objects with high reflectance reflect light brightly and appear lighter, while objects with low reflectance absorb more light and appear darker [35]. Based on the microstructure analysis of CS and PS (Figure 2), the CS particles were found to be smaller and rougher, whereas the PS particles were larger and smoother. Subsequently, the reflectance of CS was higher than that of PS. Figure 3A indicates clear absorption peaks around 700 nm for both CS-P and PS-P, suggesting a complex formation between starches and paprika red pigment. Moreover, CS-P exhibited higher reflectance compared to PS-P, resulting in a brighter and redder appearance for CS-P. The reflectivity of the complexes increased with increased CS concentration (Figure 3C), implying that a higher concentration of CS yields a redder colour for the complexes. Literature indicates that substances with high reflectivity demonstrate low scattering, and the results of this experiment confirm this hypothesis (Figure 3B,D).

### 3.3. HPLC Analysis of Adsorption of Paprika Red Pigment from CS and PS

HPLC uses column packing to selectively retain the components in the material to achieve the effect of separation of material components [36]. Moreover, HPLC is often used to detect the colourants, such as synthetic and natural colourants in green-coloured foodstuffs, curcuminoids, and synthetic edible pigments in beverages, etc. [37,38,39]. Therefore, in this study, the adsorption of CS and PS to the five pigments with the highest paprika red pigment was determined using HPLC. As shown in Table S1, the standard curve is linear (R^2^ > 0.9900). The adsorption capacity and adsorption ratio show the distribution of the substance on the adsorbent during the adsorption process. A greater adsorption capacity suggests that the adsorbent has the ability to adsorb a higher quantity of substances. A high adsorption ratio may indicate that the adsorbed substance is adsorbed effectively by the adsorbent [40]. As shown in Table 2, both CS and PS adsorbed all five pigments. However, CS adsorbed 85.44 ± 1.10 μg/g, representing 12.5 times higher adsorption capacity than PS. This result suggests that CS has superior adsorption capabilities for paprika red pigments. The adsorption ratios showed that both CS-P and PS-P had high capsanthin percentages, indicating that CS and PS could effectively adsorb capsanthin, and the red pigment is the primary colour-presenting substance in the starch-paprika red pigment complexes. Furthermore, CS had a significantly higher adsorption ratio of capsanthin than PS, implying that CS-P is redder than PS-P. Research suggests that the adsorption capacity of starch for pigment molecules is related to the amount of amylose present [41]. Therefore, it can be inferred that the variances in amylose content between CS and PS might lead to differences in paprika red pigment adsorption by these starches.

### 3.4. CI values of the Starch-Paprika Red Pigment Complexes

**Figure 4:**
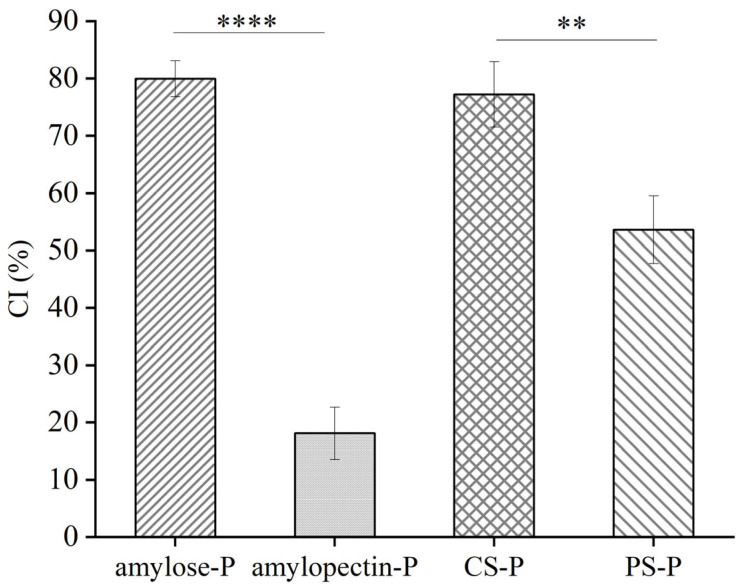
Complexing index of different starch-paprika red complexes. CS: Corn starch, PS: Potato starch, CS-P: Corn starch-paprika red pigment, and PS-P: Potato starch-paprika red pigment. Significant difference was defined as *p* < 0.05 (** *p* < 0.01, **** *p* < 0.0001). Values are expressed as mean ± standard deviation (*n* = 3).

The adsorption capacity of amylose and amylopectin for paprika red pigment was determined through the *CI* method. *CI* is based on starch-iodine complex formation in starch representing the degree of starch complexed with binding agents. The higher *CI* values indicate the enhanced degree of formation of V-type inclusion complexes between paprika red pigment and amylose molecules [42]. Herein, the adsorption capacity of CS and PS for paprika red pigment was compared, and the results showed that the capacity of CS to adsorb paprika red pigment was significantly higher than that of PS with the *CI* values of 77.23 ± 5.72% and 53.62 ± 5.90%, respectively. Secondly, the ability of amylose and amylopectin to adsorb paprika red pigment was examined, demonstrating that amylose was more readily bound to the paprika red pigment with a *CI* value of 79.97 ± 3.13%, which was 4 times more than that of amylopectin (Figure 4). This phenomenon might be attributed to the formation of starch molecules. Amylose is a primary linear polysaccharide with α-(1-4)-linked D-glucose units. The complexing agents (lipids, emulsifiers, flavor compounds, etc.) can induce amylose to form a single helix structure. The single helix structure features a notably spacious hydrophobic cavity, wherein the inner surface holds a lipophilic core comprised of C-H groups, while the outer surface consists of polar hydroxyl groups. Additionally, the lipophilic core of the single helix allows for the entrance of the aliphatic group of the complexing agent [43]. Therefore, the single helical structure of the cavity formed by the amylose could adsorb more paprika red pigment. Amylopectin consists of many short chains of glucose linked by α-1,4-glycosidic bonds, and these short chains are linked together by α-1,6-glycosidic bonds at the reducing end, making it a highly branched macromolecule. The weak complexing ability of amylopectin is attributed to the short chain lengths of amylopectin branches and their steric hindrance [44]. CS contained a considerable amount of straight-chain starch, in contrast to PS, which contained up to 79% branched-chain starch. Consequently, the *CI* of CS was significantly higher than that of PS, consistent with previous hypotheses.

### 3.5. Particle Size Analysis of Starches and Starch-Paprika Red Pigment Complexes

Particle size plays a vital role in determining the interaction between pigments and macromolecules. The average particle size of CS and PS was significantly higher than the mean particle size of the starch-paprika red pigment complexes (Table 3), which might be because the starches became gelatinous after gelatinisation during the complex formation, resulting in the adhesion between the particles and the loss of the original morphology [13]. The reduced starch particle size could be attributed to the formation of smaller composite particles during amylose release from the starch granules. Particle size can be an indicator of complex stability. Generally, the smaller the particle size, the greater the stability of the complex system [45]. The particle size of the complexes formed by starch and pigment decreased, assuming that the starch-paprika red pigment complexes were more stable. Additionally, the particle size of CS-P was smaller compared to PS-P, indicating that CS-P was more stable than PS-P. The *CI* experiment result indicated that corn starch had a strong binding ability for paprika red pigment, resulting in a higher adsorption capacity compared to PS-P. As a result, the particle size of CS-P was smaller. Similarly, it was reported that the addition of lycopene reduced the particle size of amylose, and with increasing ferulic acid concentration, the particle size of CS decreased [46]. Liang et al. fabricated nanocomplexes of chitosan hydrochloride (CHC), carboxymethyl chitosan (CMC), and anthocyanins (ACNs) through electrostatic interaction to improve the stability of ACNs. At the optimal ratio of 1.2 g CHC to 1.0 g CMC (*w*/*w*, 8 mg of ACNs), the ACNs-loaded CHC/CMC nanocomplexes showed high encapsulation efficiency with a smaller particle size [47]. This phenomenon could be attributed to the fact that after the starches interact with the small molecule, water is expelled from the complex in the presence of the hydrophobic cavity.

### 3.6. Pore Analysis of Starches and Starch-Paprika Red Pigment Complexes

The pore structure affects the adsorption properties, solubility, and storage of substances. The pore area represents a substance’s internal pore structure and helps evaluate its adsorption properties. Porosity is a useful tool for determining the porosity and permeability of a substance [48]. Generally, substances with large pore areas and high porosity exhibit more adsorption sites, resulting in an increased ability to adsorb the targeted substances effectively. Starch has a certain pore structure, so studying and evaluating the pore area and porosity of starch are imperative to expand the starch application and modification. According to Table 3, CS displayed greater pore area and porosity than PS. This result can be explained by the microstructure of starch. As shown in Figure 2, the PS granules had a smooth surface with no signs of pores and cracks. In contrast, the CS particles had more surface pores and cracks, endowing them with better adsorption properties. Therefore, CS-P was redder than PS-P. Lower porosity improves the substance’s stability due to the reduced number of voids and pores within the substance. This reduction limits the pathways for air and moisture to enter and contributes to a reduction in some reactions, including oxidation, decomposition, and deterioration [49]. The porosity of CS-P was significantly lower than that of CS after the complexation of starch pigment. However, there was no significant difference between the porosity of PS-P and PS. These results suggest that the inclusion of paprika red pigment improved the stability of CS but had minimal impact on the stability of PS. This experimental result was consistent with the results of *CI* and particle size analyses. Su et al. found that the porosity of tapioca starch decreased following the complexation of pigments from sugarcane juice with tapioca starch [50]. These results offer theoretical guidance for developing an effective adsorbent.

### 3.7. Structural Characterisation of Starchs and Starch-Paprika Red Pigment Complexes

#### 3.7.1. FT-IR Spectroscopy Analysis

**Figure 5:**
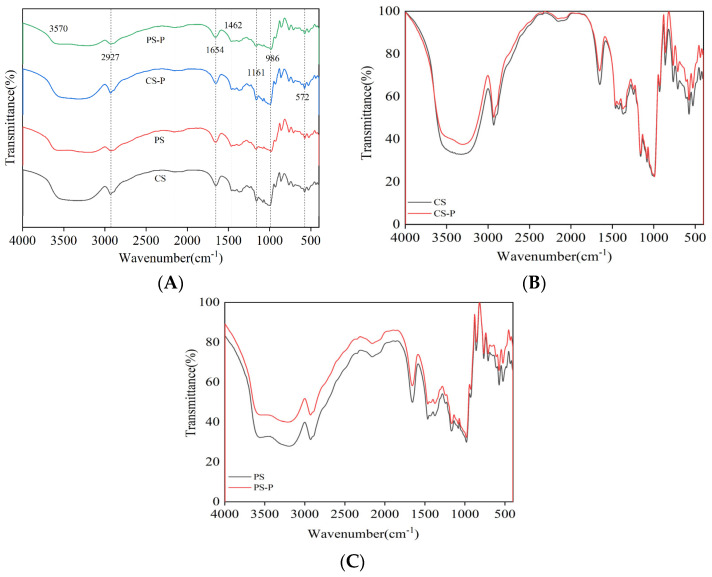
FT−IR spectra of starches and starch−paprika red complexes (**A**), Green line: PS−P, Blue line: CS−P, Red line: PS, Black line: CS. FT−IR spectra of CS and CS−P complex (**B**), and FT−IR spectra of PS and PS−P complex (**C**) are shown. CS: corn starch, PS: potato starch, CS−P: corn starch−paprika red complex, and PS−P: potato starch−paprika red complex.

FT-IR spectra are primarily used to observe the differences in the crystalline and amorphous regions between the complexes and the natural starch and the bonding forces [51]. Normally, starch shows typical bands and peaks in FT-IR spectra. Therefore, in this study, the effect of paprika red pigment on the structure of starch was explored using FTIR spectroscopy. The infrared spectra of CS, PS, CS-P, and PS-P complexes are illustrated in Figure 5. The typical common absorption peaks are as follows: The absorption peak near 572 cm^−1^ refers to the skeletal mode vibration of starch, the absorption peak at 986 cm^−1^ refers to the backbone vibration of the asymmetric ring mode (α-1,4-glycosidic bonds (C-O-C) of starch, the absorption peaks at 1161 cm^−1^ refers to the C-O bond and the stretching vibration of the C-C bond, the band at 1462 cm^−1^ refers to the bending vibration of CH2 [52], and the band around 1654 cm^−1^ refers to the absorption peak in the amorphous region of adsorbed water in starch. The band at 2156 cm^−1^ was probably due to the presence of free water in starch. An intermediate intensity peak at 2930 cm^−1^ refers to the antisymmetric stretching oscillation of CH2, and the band around 3000 cm^−1^ to 3600 cm^−1^ was dominated by hydrogen bond stretching vibrations and absorption, which was a sensitive indicator to characterize the strength of hydrogen bonds [53]. As shown in Figure 5A, no new absorption peaks appeared, and no absorption peaks disappeared for the starch-paprika red pigment complex compared to CS and PS, indicating no chemical bond modification or covalent bond formation between starch and paprika red pigment. Overall, the addition of paprika red pigment had little effect on the chemical bonding of CS and PS molecules. As shown in Figure 5B,C, after the addition of paprika red pigment, the absorption peaks of CS-P and PS-P complexes were enhanced at 3421.82 cm^−1^, indicating the formation of a strong hydrogen bond between starches and paprika red pigment [54]. 

#### 3.7.2. X-Diffraction Analysis

**Figure 6:**
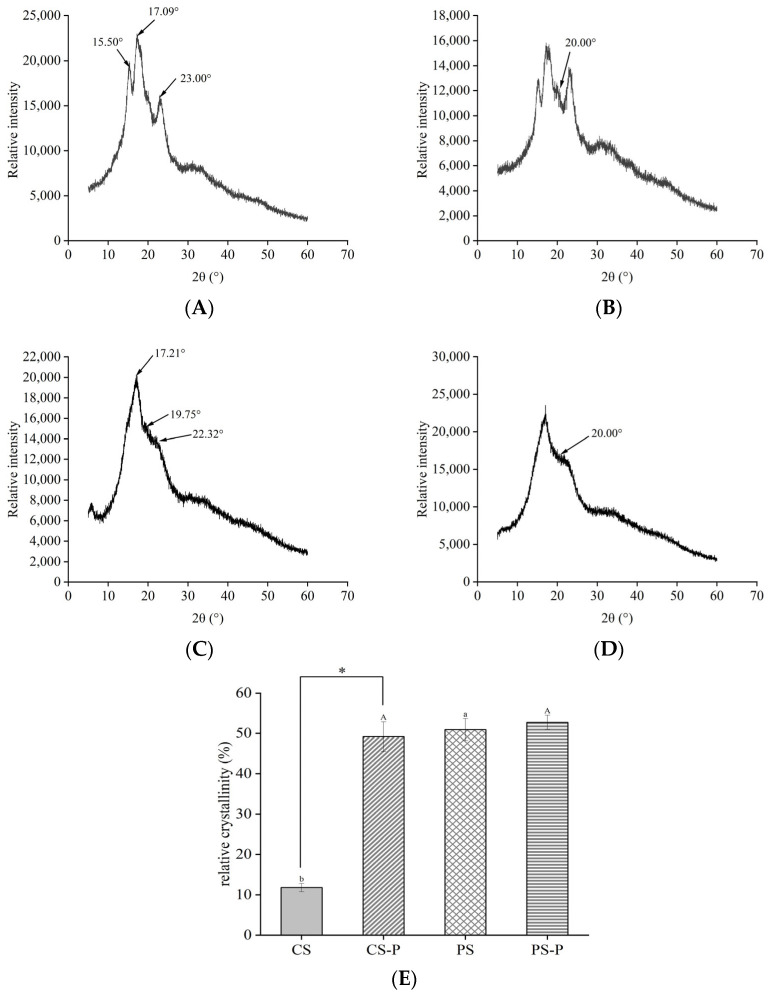
X–ray diffraction of starchs and starch–paprika red complexes. (**A**–**D**) represent CS, CS-P, PS, and PS-P, respectively. The relative crystallinity of starches and starch–paprika red pigment complexes (**E**) is shown. CS: corn starch, PS: potato starch, CS-P: corn starch–paprika red complex, and PS-P: potato starch–paprika red complex. * indicates a significant difference between CS and CS-P (*p* < 0.05). Different small letters indicate significant differences between CS and PS (*p* < 0.05). Different capital letters represent significant differences between CS-P and PS-P (*p* < 0.05).

XRD plays a crucial role in identifying the formation of starch complexes with polyphenols, lipids, and anthocyanins [55]. Therefore, in this study, the binding mechanism between starches and paprika red pigment was determined via XRD. The XRD analysis revealed that type A starch exhibited notable peaks at 2θ = 15.05°, 17.09°, 17.92°, and 23.00° while type B starch exhibited discernible absorption peaks at 2θ = 15.26°, 17.21°, 19.75°, 22.32°, and 24.08° [56]. As shown in Figure 6A,C, CS exhibited strong characteristic peaks at 2θ = 15.50°, 17.09°, and 23.00° and PS exhibited characteristic peaks at 2θ = 17.21°, 19.75°, and 22.32°, indicating that CS exhibits a typical A-type diffraction peak while PS displays a typical b-type diffraction peak. Additionally, the starch V-shaped structure showed characteristic broad peaks at 13.00° and 20.00°. The CS-P and PS-P complexes showed characteristic broad peaks at 20.00° (Figure 6B,D). It can be seen that the starch formed a V-shaped structure after binding to the paprika red pigment, which was consistent with the findings of Liu et al. The presence of paprika red pigment significantly altered the crystal structure of starch. A higher proportion of ligand material increases the probability of forming a V-shaped structure [57]. As shown in Figure 6E, CS-P had a relative crystallinity of 49.21 ± 3.67%, which was significantly higher than that of CS with 11.77 ± 0.99%. Additionally, PS-P had a slightly higher relative crystallinity of 52.74 ± 1.75% than that of PS with 50.93 ± 2.74%. These results suggested that the resultant structure was more stable and compact, with increased relative crystallinity due to the complexation of starch with paprika red pigment.

## 4. Conclusions

In summary, the CS-P and PS-P complexes were synthesized using CS, PS, and paprika red pigment, and their structures were analyzed. The results showed that both CS and PS had the ability to adsorb paprika red pigment, with CS exhibiting the highest ability. CS-P exhibited a brighter and redder appearance than PS-P. The particle size and pore structure analyses demonstrated that the stability of the starch-paprika red pigment complexes was surpassed compared to the starch molecules. Moreover, CS-P displayed greater stability than PS-P. The FT-IR and XRD analyses demonstrated that strong hydrogen bond interactions were formed between the starch molecules and paprika red pigment. The starch-paprika red pigment complexes displayed a V-type structure with increased relative crystallinity. However, the relative crystallinity of CS-P was greater than that of PS-P, suggesting that the stability of CS-P was superior to that of PS-P. Overall, CS-P has a better colour appearance than PS-P. This study provides a theoretical basis for further exploitation of paprika red pigment and starch. In the future, the starch-pigment complexes can be used in the delivery systems to develop more optimal controlled-release and targeted drug delivery systems. Despite the positive outcomes, the study has many limitations that need to be addressed. Firstly, the colour difference in capsanthin in different starches is influenced by various factors, such as the properties of starch particles, the optical properties of the dispersion system, and the selective adsorption of red and yellow pigments in capsaicin. However, quantifying the contribution of each factor remains a challenge. In addition, further investigation into the colouring effect of different starches on capsanthin is imperative.

## Figures and Tables

**Table 1:** Values of colour indices L*, a*, b*, h and ΔE of CS-P, PS-P and CS-P-SC complexes.

	L*	a*	b*	ΔE
PS-P	42.63 ± 0.41 ^b^	22.45 ± 1.84 ^b^	20.83 ± 1.40 ^b^	6.75 ± 0.58
CS-P	46.01 ± 0.41 ^a^	26.90 ± 0.23 ^a^	25.39 ± 0.50 ^a^	12.57 ± 0.63 ***
CS-P-SC	38.80 ± 0.11 ^c^	18.44 ± 0.67 ^c^	15.69 ± 0.47 ^c^	-

ΔE denotes the total colour difference calculated using the CS-P-SC dispersion system as a control. CS-P: Corn starch-paprika red pigment, PS-P: Potato starch-paprika red pigment, and CS-P-SC: Corn starch-paprika red pigment-sodium caseinate. Values with different letters in the same column are significantly different (*p* ≤ 0.05). Significant difference was defined as *p* < 0.05 (*** *p* < 0.001). Values are expressed as mean ± standard deviation (*n* = 3).

**Table 2:** Adsorption analysis of carotenoids in paprika red pigment by CS and PS.

		Adsorption Capacity (μg/g)	Adsorption Ratio/%
CS	capsorubin	3.56 ± 0.03 ^e^	4.57 ± 0.50 ^d^
	capsanthin	40.63 ± 0.65 ^a^	48.24 ± 1.79 ^a^
	zeaxanthin	6.50 ± 0.08 ^d^	8.78 ± 1.37 ^cd^
	β-cryptoxanthin	10.00 ± 0.15 ^c^	11.85 ± 1.87 ^c^
	β-carotene	24.75 ± 0.19 ^b^	29.81 ± 4.84 ^b^
	Total	85.44 ± 1.10	100
PS	capsorubin	0.40 ± 0.01 ^d^	7.37 ± 1.20 ^c^
	capsanthin	2.75 ± 0.25 ^a^	42.61 ± 2.94 ^a^
	zeaxanthin	0.48 ± 0.08 ^cd^	9.09 ± 1.90 ^c^
	β-cryptoxanthin	0.75 ± 0.06 ^c^	11.60 ± 2.24 ^c^
	β-carotene	2.27 ± 0.30 ^b^	34.14 ± 5.36 ^b^
	Total	6.65 ± 1.53	100

CS: Corn starch, PS: Potato starch. Different letters indicate significant differences (*p* < 0.05). Values (mean ± SD) were calculated using the results from three independent experiments.

**Table 3:** Particle size, pore area, and porosity of starches and starch-paprika red pigment complexes.

	Particle Size (µm)	Pore Area (m^2^)	Porosity (%)
CS	10.16 ± 0.12 ^b^	0.48 ± 0.06 ^a^	55.44 ± 0.65 ^a^
PS	27.54 ± 0.28 ^a^	0.19 ± 0.05 ^b^	40.44 ± 0.75 ^c^
CS-P	5.27 ± 0.60 ^c^	0.48 ± 0.02 ^a^	46.05 ± 2.12 ^b^
PS-P	26.34 ± 0.22 ^a^	0.25 ± 0.06 ^b^	41.63 ± 1.81 ^c^

CS: Corn starch, PS: Potato starch, CS-P: Corn starch-paprika red pigment, and PS-P: Potato starch-paprika red pigment. Values with different letters in the same column are significantly different (*p* ≤ 0.05). Values (mean ± SD) were calculated using the results from three independent experiments.

## Data Availability

All original data included in this paper are available from the authors upon reasonable request. The data are not publicly available due to privacy.

## Supplementary Materials

The following supporting information can be downloaded at: https://www.mdpi.com/article/10.3390/foods13020191/s1, Table S1: Standard curves for five pigments.
