# Supplementary material for: Differences in the Chromogenic Effect of Corn Starch and Potato Starch on Paprika Red Pigment and Structural Characterisation
Source: Foods. 2024 Jan 6;13(2):191. doi: 10.3390/foods13020191 (PMC10814249; doi:10.3390/foods13020191)
Supplement: Supplementary file 1 [file foods-13-00191-s001.zip › foods-2772353-supplementary.pdf]

Table S1. Standard curves for five pigments

Pigment name	Equation	R^2
capsorubin	y=1203.2x+218.35	0.9982
capsanthin	y=1730.7x-212.89	0.9999
zeaxanthin	y=2417.6x+1495.3	0.9922
β-cryptoxanthin	y=2123.5x-306.77	0.9992
β-carotene	y=974.44x-174.02	0.9995
